# SubID, a non-median dichotomization tool for heterogeneous populations, reveals the pan-cancer significance of *INPP4B* and its regulation by EVI1 in AML

**DOI:** 10.1371/journal.pone.0191510

**Published:** 2018-02-07

**Authors:** Irakli Dzneladze, John F. Woolley, Carla Rossell, Youqi Han, Ayesha Rashid, Michael Jain, Jüri Reimand, Mark D. Minden, Leonardo Salmena

**Affiliations:** 1 Princess Margaret Cancer Centre, University Health Network, Toronto, Canada; 2 Department of Medical Biophysics, University of Toronto, Toronto, Canada; 3 Ontario Institute of Cancer Research, Toronto, Canada; 4 Department of Pharmacology and Toxicology, University of Toronto, Toronto, Canada; Wayne State University, UNITED STATES

## Abstract

Our previous studies demonstrated that INPP4B, a member of the PI3K/Akt signaling pathway, is overexpressed in a subset of AML patients and is associated with lower response to chemotherapy and shorter survival. *INPP4B* expression analysis in AML revealed a right skewed frequency distribution with 25% of patients expressing significantly higher levels than the majority. The 75% low/25% high cut-off revealed the prognostic power of *INPP4B* expression status in AML, which would not have been apparent with a standard median cut-off approach. Our identification of a clinically relevant non-median cut-off for *INPP4B* indicated a need for a generalizable non-median dichotomization approach to optimally study clinically relevant genes. To address this need, we developed Subgroup Identifier (SubID), a tool which examines the relationship between a continuous variable (e.g. gene expression), and a test parameter (e.g. CoxPH or Fisher’s exact *P* values). In our study, Fisher’s exact SubID was used to reveal EVI1 as a transcriptional regulator of *INPP4B* in AML; a finding which was validated *in vitro*. Next, we used CoxPH SubID to conduct a pan-cancer analysis of INPP4B’s prognostic significance. Our analysis revealed that *INPP4B*^low^ is associated with shorter survival in kidney clear cell, liver hepatocellular, and bladder urothelial carcinomas. Conversely, *INPP4B*^low^ was shown to be associated with increased survival in pancreatic adenocarcinoma in three independent datasets. Overall, our study describes the development and application of a novel subgroup identification tool used to identify prognostically significant rare subgroups based upon gene expression, and for investigating the association between a gene with skewed frequency distribution and potentially important upstream and downstream genes that relate to the index gene.

## Introduction

Given the high degree of inter- and intra-tumor heterogeneity present in cancer, there is a great need for effective patient stratification strategies that will direct specific patient subpopulations to optimal therapies. An effective stratification strategy would consider the genetic, molecular, and cellular landscapes of specific tumours and classify them as sensitive or resistant to a given therapy or treatment regimen. If implemented effectively, such a strategy will likely result in the increased success of available therapies, and certainly increase the success of clinical trials. Indeed, numerous efforts have already identified important subgroups within cancers based on defined genetic and molecular parameters and morphology. For example, in AML Valk *et al*. identified 16 distinct AML subgroups as defined by key molecular signatures based on high throughput microarray gene expression analysis of 285 AML patients [1]. Similar analysis in pancreatic patients revealed four defined subtypes [2]. Breast cancers are classified based on molecular features that include the expression or overexpression of growth factor receptors such as the Erb-B2 receptor tyrosine kinase 2 (ERBB2/HER2), ER, and PgR [3]. Identification of these features has guided the development of receptor tyrosine kinase inhibitors for the treatment of HER2 positive breast cancer subtype, demonstrating the translational value of informed disease stratification.

Despite recent advances in characterizing the genomic landscape of cancers, we are still unable to accurately predict patient survival following specific therapies, nor identify the cancer driving abnormalities in most patients. Herein, we tackle both these problems through the development of a tool for identifying and studying subgroups within heterogeneous populations. Herein, we describe the development and application of SubID and use the expression of *Inositol Polyphosphate 4-Phosphatase type II (INPP4B)* as a representative gene expression-based biomarker for predicting patient survival across cancers.

INPP4B is a phospholipid phosphatase that catalyzes the removal of a D4 phosphate group from PI(3,4)P_2_ to generate PI(3)P [4,5]. INPP4B was first isolated and identified as a 105 kDa D4 lipid phosphatase by Norris *et al*. who demonstrated that INPP4B dephosphorylated PI(3,4)P_2_ to PI(3)P >900-fold more efficiently compared to its impact on PI(1,3,4)P_3_ (4). PI(3,4)P_2_, along with PI(3,4,5)P_3_, have been proposed to be necessary for full activation of the pro-survival kinase AKT [6,7].

In keeping with its role in limiting pro-survival signaling through AKT, INPP4B was proposed to be a putative tumor suppressor [8–10]. Studies in breast, prostate, melanoma and ovarian cancers provided evidence for the tumor suppressive role of INPP4B [9,11–13]. In human breast cancer models, INPP4B suppressed epithelial cell transformation, proliferation and anchorage-independent growth in an AKT-dependent manner [9,10]. Similarly, INPP4B overexpression in prostate cancer cells decreased levels of phosphorylated AKT, and decreased cellular invasion [11]. Clinically, loss of INPP4B expression in breast cancer was associated with decreased patient survival. In particular, LOH at the *INPP4B* locus frequently occurred in basal-like (triple negative) breast cancers, characterized by poor clinical outcome [8,9,14,15]. Furthermore, Won *et al*. demonstrated INPP4B to be a biomarker of basal-like breast cancer subtype with 99% specificity and 61% sensitivity [15]. Similarly, loss of *INPP4B* in ovarian cancer was associated with shorter OS and higher rates of lymph node metastasis [9,13].

In contrast to its reported role in tumor suppression, emerging evidence suggest that high levels of INPP4B may promote the development and progression of some cancers. For instance, INPP4B can promote the growth and proliferation of ER^+^ breast cancer cells through SGK-3, a family member of serine/threonine kinases closely related to AKT [16]. In addition, two independent studies in AML reported that *INPP4B* overexpression is associated with poor clinical outcome and chemotherapy resistance [17,18]. In colon cancer cells, INPP4B overexpression was shown to be associated with increased proliferation, anchorage independent growth and xenograft growth through a mechanism which involves destabilization of PTEN [19].

In melanoma, one study reported that low levels of INPP4B expression was associated with tumor progression, while another study reported a SGK3-dependent oncogenic role for INPP4B in a high expressing subset of the same disease [12,20]. Moreover, in a *PTEN*-deficient model of thyroid malignancy, INPP4B was proposed to compensate for PTEN loss by dephosphorylating PTEN substrates such as PI(3,4,5)P_3_ [21]. This seemingly paradoxical association of INPP4B expression with cancer suggests that the role of INPP4B in cancer is cell type- or context-dependent. Importantly, these observations point to the need of ascertaining the genetic and molecular background of the host disease to predict contextual consequence of INPP4B activity.

Despite emerging studies, much remains unclear about the consequences of differential *INPP4B* expression in cancer including: how expression is regulated and the relationship between *INPP4B* expression levels and patient outcome. Our previous work in AML showing that *INPP4B* is highly expressed and associated with adverse outcome in 25% of AML patients [17] exemplified the value of a non-median dichotomization approach and recognized the existence of small, prognostically distinct subgroups within a population. We propose that non-median dichotomization is more appropriate than median dichotomization in the assessment of clinical biomarkers. Thus we introduce a novel subgroup identification tool named Subgroup Identifier (SubID) and propose that *INPP4B* and its context dependency could provide a compelling example to illustrate its utility.

## Materials and methods

### Gene expression and clinical data

Genome-wide expression data and respective clinical information was downloaded from The Cancer Genome Atlas (TCGA; https://tcga-data.nci.nih.gov/tcga/) for datasets with n>100 patients. RNAseq data were used where available (23/25 datasets), while microarray data was used alternatively (GBM and OV datasets). Normalized microarray data for the Verhaak (GSE6891) and Chen (GSE57495) datasets were downloaded from GEO database (http://www.ncbi.nlm.nih.gov/geo/) (1). ICGC pancreatic cancer data was downloaded from the ICGC database (http://icgc.org/).

### Subgroup Identifier (SubID)

**Fig 1:**
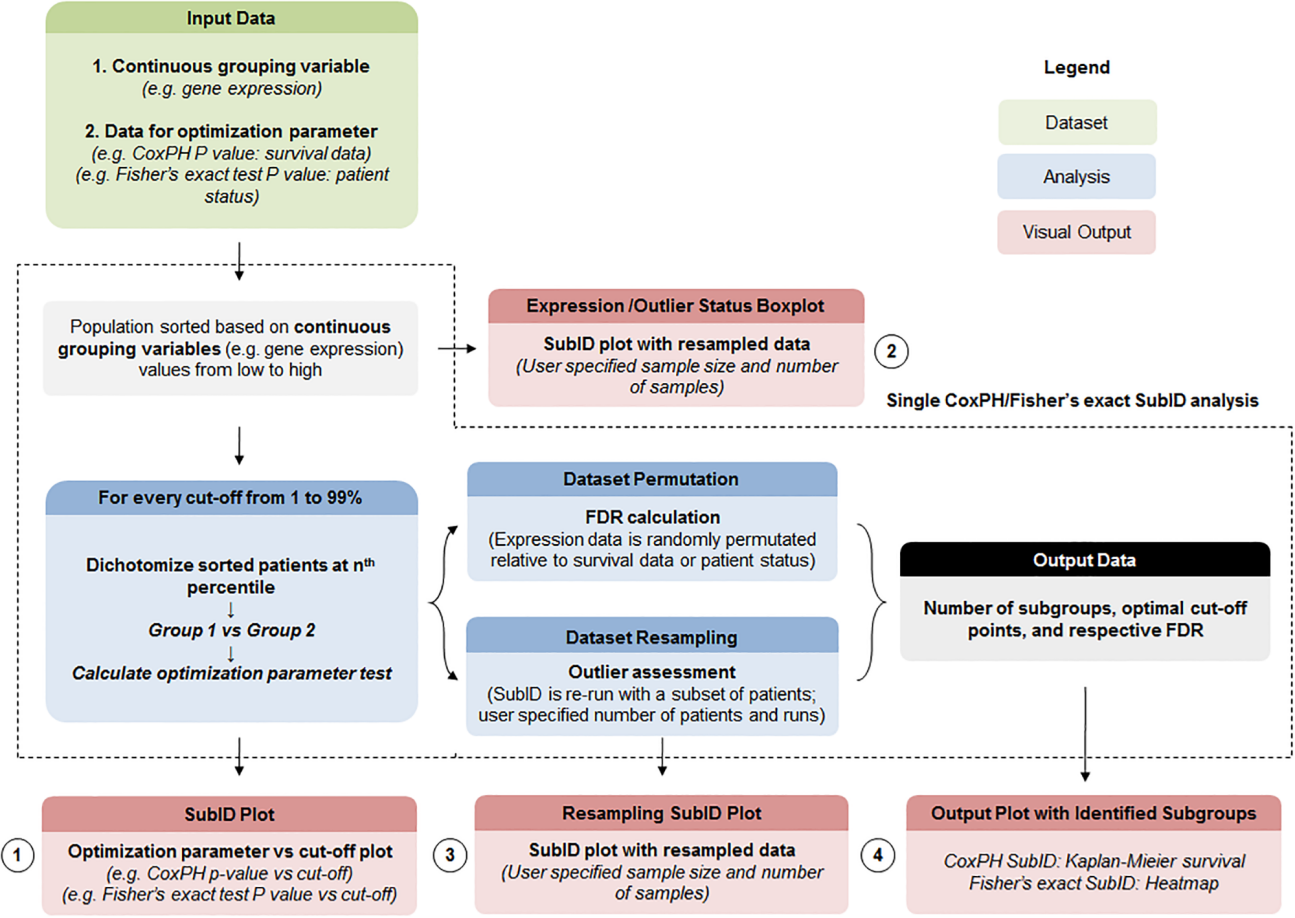
SubID pipeline. Input containing continuous grouping variable levels and data necessary for calculating an optimization parameter is loaded into SubID (R script), and the population is sorted based on levels of the continuous variable. Next, the population is dichotomized at every percentage from 1 to 99%, and the optimization parameter test is calculated at each cut-off. Permutation and resampling analysis is used to assess FDR. SubID pipeline standard output includes (1) SubID plot, (2) Boxplot depicting levels of continuous variable with outlier status (3) Resampled SubID plot and (4) output plot appropriate for the optimization parameter.

SubID was developed and used in R (https://www.r-project.org/). The input for SubID is a spreadsheet which includes a continuous variable (eg. gene expression) and necessary data for calculating the optimization parameter used for subgroup identification (Fig 1). In this study, optimization parameters included Fisher’s exact test *P* value (grouping data required) and CoxPH *P* value (OS time and status required), however other tests can be used as needed. Once input data is loaded, SubID sorts the population in order from low to high value of the continuous variable. Next, a loop is initiated which calculates the specified test (optimization parameter) comparing the low and high continuous variable value groups dichotomized at every percentage point from 1 to 99%. The results from this loop are saved into an output file, and plotted as a percentage cut-off (1 to 99%) vs optimization parameter test result plot (SubID plot). This plot visualizes the relationship between the continuous variable and the optimization parameter across the population. A subset of the patients (70% of population) is then sampled 100 times without replacement to assess and visualize the effect of outlier patients (resampled SubID plot). Mean absolute deviation of greater than two is calculated and visualized on the resampled SubID plot to examine association with expression distribution. Furthermore, resampling provides an indication as to how many reoccurring dichotomization points were present in the population which corresponded to the number of distinct subgroups. The data was also permutated 1000 times to calculate the false discovery rate (FDR) of the optimization parameter test result. Both FDR at the optimal cut-off point (FDR_A.C._) and desired cut-off range (FDR_range_) were calculated. FDR_A.C._ regulates likelihood that *P* value at cut-off is better than one observed by chance, while FDR_range_ regulates likelihood that any significant cut-off within the desired range is not by chance. Significant dichotomization points were applied to the population and visualized using appropriate plots. Though primarily designed to study a single continuous variable, SubID was also modified for a high-throughput application to identify continuous variables matching specified criteria. For this application, the SubID core process was repeated for every continuous variable in the dataset. This process generated a continuous variable vs percentage cut-off optimization parameter result table. The output data can then be filtered based on specified criteria as required.

### Expression-based subgrouping (CoxPH SubID)

To examine the prognostic value of *INPP4B* expression across cancers, *INPP4B* expression and survival data for each cancer was loaded into SubID. Following sorting based on expression, CoxPH was calculated for every dichotomization point from 1 to 99% comparing survival in *INPP4B*^low^ vs *INPP4B*^high^ subgroups. However, to avoid extremely small subgroups which would interfere with meaningful subsequent analysis, only optimal cut-offs from 10 to 90% were selected. Following resampling and permutation, the significant cut-offs were applied to the population and the survival data was visualized using a Kaplan-Meier survival plot. As indicated by the resampling plot, the population was dichotomized into either two (*INPP4B*^low^ and *INPP4B*^high^) or three (*INPP4B*^low^, *INPP4B*^inter^ and *INPP4B*^high^) subgroups as appropriate (dependent on number of reoccurring cut-off clusters). A hazard ratio (HR) relative to *INPP4B*^low^ expression was calculated.

### Co-expression analysis (Fisher’s Exact SubID)

To identify genes co-expressed with *INPP4B*, whole genome expression data and *INPP4B* expression status (*INPP4B*^low^ indicated with 0, and *INPP4B*^high^ indicated with 1) were loaded into SubID. A high-throughput version of SubID was then applied: the single-gene SubID pipeline was applied to every gene, calculating the Fisher’s exact test *P* value for every percentage cut-off (*gene*^low^ vs *gene*^high^ Fisher’s exact test looking at association with *INPP4B*^low^ vs *INPP4B*^high^). An output table with genes vs Fisher exact test *P* value across cut-offs was generated. To establish an *INPP4B*^high^ signature in AML (optimal *INPP4B* expression cut-off at 75%), genes with optimal cut-offs at 75±15% were sorted based on their optimal *P* value. DNA binding transcription factors were selected out of that list as potential regulators of *INPP4B* expression. *INPP4B*^high^ signature was visualized in Gene Set Enrichment Analysis (GSEA) tool from the Broad Institute (http://software.broadinstitute.org/gsea/index.jsp).

### Survival, univariate and multivariate analysis

Kaplan-Meier survival analysis was used to compare survival between patients dichotomized based on *INPP4B* expression status at the optimal cut-off(s) as described above. CoxPH model was used to calculate the associated HR and respective 95% confidence interval (CI). Univariate analysis was used to identify any association between *INPP4B* expression status and other clinical features. Fisher’s exact test was used for categorical data, and Wilcoxon rank-sum tests for continuous data. CoxPH model was used to calculate the OS rates. For multivariate analysis, a preliminary model was constructed for using covariates with a univariate *P*<0.05. Reverse selection (based on Akaike information criteria; AIC) was used to remove covariates and establish the optimal multivariate model for OS.

### Cell culture

*EVI1*^low^ U937 and *EVI1*^high^ OCI/AML-4, OCI/AML-6, and UCSD-AML-1 cell lines were cultured in alpha minimal essential medium (AMEM) supplemented with 10% fetal bovine serum, 100 units/ml penicillin, 100 ug/ml streptomycin, and 10% 5637 conditioned media at 37°C and 5% CO_2_. All OCI-AML series cell lines were generated in the lab of MDM. UCSD-AML-1 was purchased from the Leibniz Institute DSMZ-German Collection of Microorganisms and Cell Cultures (Braunschweig, Germany).

### Viral infections

EVI1 cDNA was first cloned into the MSCV-puro-IRES-GFP (PIG) retroviral construct [17]. Briefly, MSCV-PIG and appropriate packaging constructs were transfected into 293T using the calcium phosphate method; at 48 and 72 hours following transfection viral particle-rich supernatant was collected and concentrated with Lenti-X concentrator (Clonetech, Mountain View, CA, USA). Next, U937, OCI-AML-3, OCI-AML-4 and UCSD/AML-1 cells were infected with virus for 24h in the presence of 8ug/ml protamine sulfate and followed by puromycin selection for 48h.

### Quantitative RT-PCR

RNA was extracted from cells using RNeasy Mini kit (Qiagen) and first-strand cDNA synthesis was performed using the MMLV systems (Life Technologies). qPCR was performed in triplicate using SYBR-Green PCR Master Mix kit (Applied Biosystems) on a 7900 HT Real-Time PCR system with SDS v2.3 software (Applied Biosystems) using standard settings: 95°C (10 min) and 40 cycles of 95°C (20 sec) and 60°C (1 min). mRNA levels were normalized to *β2M* housekeeping gene. The following primers were used for qPCR: *EVI1* 5’- CTTCTTGACTAAAGCCCTTGGA-3’ (sense) and 5’- GTACTTGAGCCAGCTTCCAACA-3’ (antisense); *INPP4B* 5’-GGAAAGTGTGAGCGGAAAAG-3’ (sense) and 5’-CGAATTCGCATCCACTTATTG’-3’ (antisense); *β2M* (control), 5’-TGCTGTCTCCATGTTTGATGTATCT-3’ (sense) and 5’-TCTCTGCTCCCCACCTCTAAGT-3’ (antisense).

### Chromatin immunoprecipitation

Chromatin immunoprecipitation was done with anti-EVI1 (#2593), IgG (#7076) and H3 (#9715) antibodies from Cell Signaling Technology (CST, Danvers, MA, USA) with the EZ-ChIP kit (#17–371) from Millipore (Etobicoke, ON, Canada) as directed. EVI1 binding enrichment at the +1379bp and -1000bp (negative control) sites was examined using quantitative PCR as previously described (Dzneladze et al., 2015). The following primers were used: EVI1 +1379bp 5’-TACCTTTGAACGGCTCCATC-3’ (sense) and 5’- CCCACTTCCTAGCCCCTAAC-3’ (antisense); EVI1 -1000bp 5’- TACTGGAAAACCCGGTAGG-3’ (sense) and 5’- CTGACAGGAAGGAGATATGCAA-3’ (antisense).

## Results

### SubID development and testing in AML

**Fig 2:**
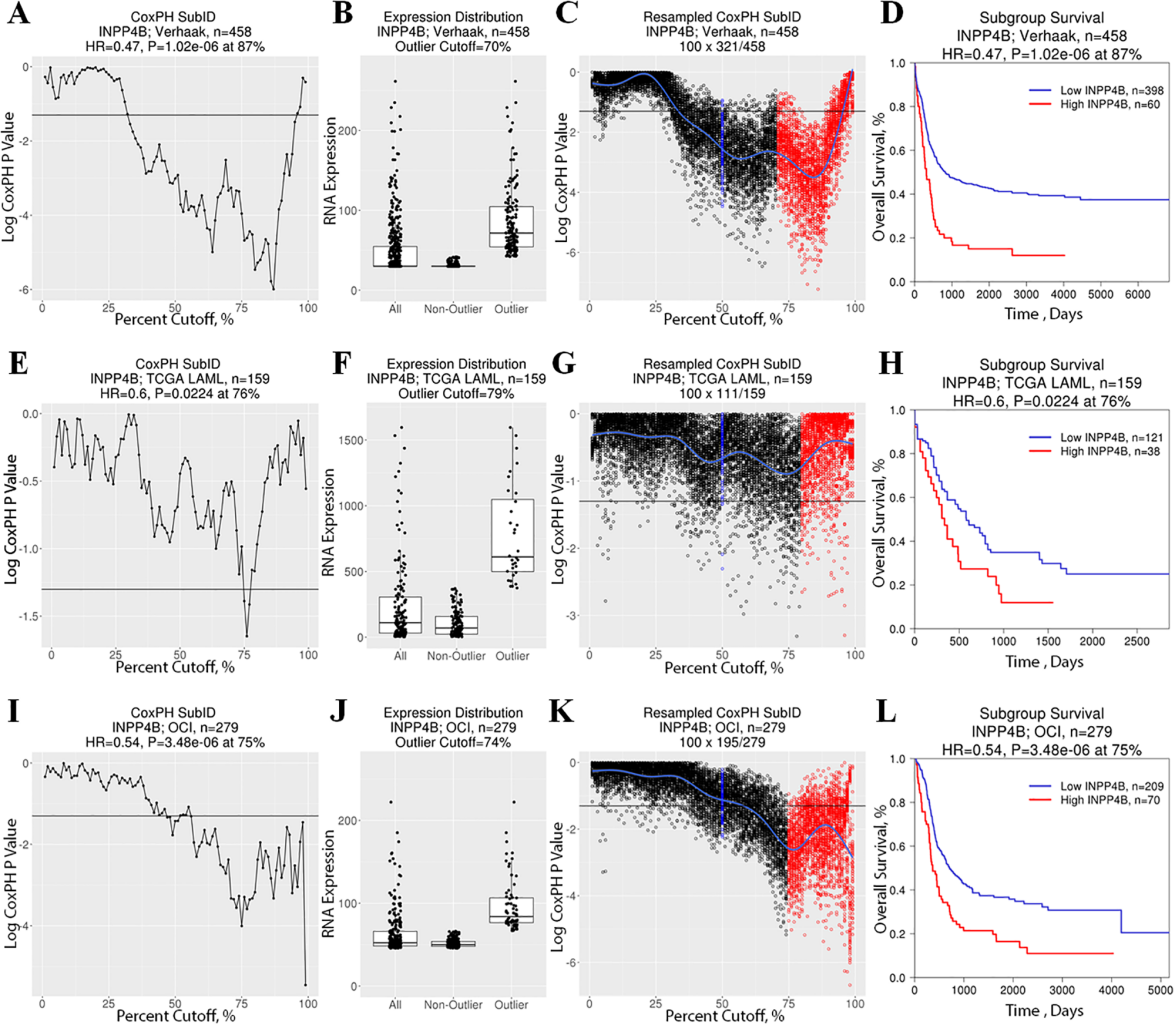
SubID application to *INPP4B* across AML datasets. Standard CoxPH SubID output consisting of a (A,E,I) SubID plot, (B,F,J) *INPP4B* expression boxplot with expression distribution outlier status, and (C,G,K) resampled SubID plot with median (blue) and outlier cut-off values (based on expression distribution; red) for the Verhaak, TCGA and OCI AML datasets, respectively. (D,H,L) Kaplan-Meier plot of patients dichotomized at the SubID optimal cut-off demonstrates a significant survival difference between *INPP4B*^low^ and *INPP4B*^high^ subgroups.

In order to demonstrate its functionality in optimizing population dichotomization with known or candidate biomarkers, we used CoxPH SubID to validate the previously reported relationship between *INPP4B* expression levels and patient outcome in AML. Examination of the prognostic significance of *INPP4B* in the Verhaak, TCGA and OCI/PM AML datasets with CoxPH SubID identifies optimal cut-offs for *INPP4B* at 87% in the Verhaak dataset (*P* = 1.02E-06, median cut-off *P* = 9.77E-04), 76% in the TCGA dataset (*P* = 0.0224, median cut-off *P* = 0.359), and 75% in the OCI/PM dataset (*P* = 6.48E-06, median cut-off *P* = 0.0373) (Fig 2A, 2E and 2I). As previously reported, gene expression analysis revealed that *INPP4B* is overexpressed in 21–30% of AML patients (Fig 2B, 2F and 2J; S1 Fig). Resampling analysis demonstrates that the optimal *INPP4B* cut-off point coincides with the *INPP4B*^high^ expression status thereby suggesting a prognostically significant role of *INPP4B* overexpression (Fig 2C, 2G and 2K).

SubID validates our previous results showing that patients dichotomized based *INPP4B* expression status into *INPP4B*^low^ and *INPP4B*^high^ subgroups exhibit significantly different patient survival (Fig 2D, 2H and 2L). Specifically, *INPP4B*^high^ AML is associated with significantly shorter overall patient survival compared to *INPP4B*^low^ patients. Furthermore, survival analysis reveals an improved risk stratification of AML patients based on SubID subgrouping into *INPP4B*^high^ and *INPP4B*^low^ groups as compared to median dichotomization (Fig 2D, 2H and 2L; S2 Fig). Together, these findings demonstrate that CoxPH SubID can improve upon median dichotomization to identify distinct patient subpopulations based on gene expression of a prognostically informative gene.

### Fisher’s exact SubID identifies transcriptional regulators of *INPP4B* expression

**Fig 3:**
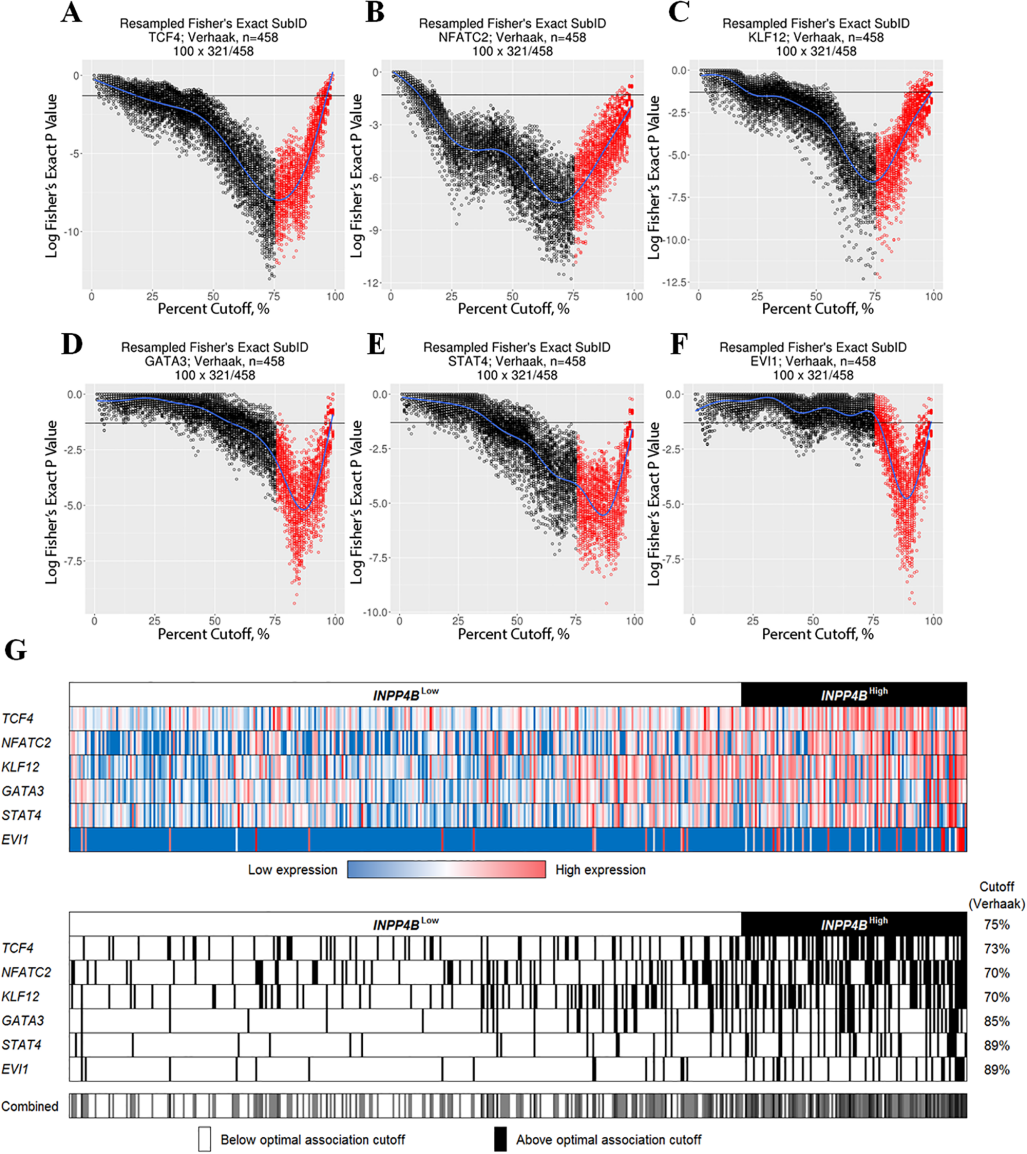
Transcriptional regulators of *INPP4B* (Verhaak dataset). (A-F) resampled Fisher’s exact SubID plot for the top six DNA-binding transcription factors co-expressed with *INPP4B* in Verhaak dataset. (G) Color and status (based on Verhaak dataset-derived maximal co-expression analysis) heatmaps of the top potential transcriptional regulators of *INPP4B* expression in AML (Verhaak dataset).

Following initial testing and validation, SubID was used to build upon our previous characterization of *INPP4B* in AML [17]. Specifically, we sought to identify transcriptional regulators of *INPP4B* to explain the observed overexpression in 25% of AML patients. For this, we first used high-throughput Fisher’s exact SubID analysis of the Verhaak AML dataset to identify transcription factors significantly co-expressed with *INPP4B*. Specifically, the non-median dichotomization approach used in SubID allowed for selection of transcription factors that have optimal association with *INPP4B*^high^ with a 75% cut-off. Transcription factors with the most significant co-expression with *INPP4B* in the Verhaak dataset included *TCF4* (73% cut-off, *P* = 4.5E-13), *NFATC2* (70% cut-off, *P* = 1.1E-11), *KLF12* (70% cut-off, *P* = 1.4E-09), *GATA3* (85% cut-off, *P* = 4.9E-09), *STAT4* (89% cut-off, *P* = 6.6E-08) and *EVI1* (89% cut-off, *P* = 3.1E-08). (Fig 3). Candidate *INPP4B* transcription factors were cross-validated in the TCGA LAML dataset (S3 Fig).

**Fig 4:**
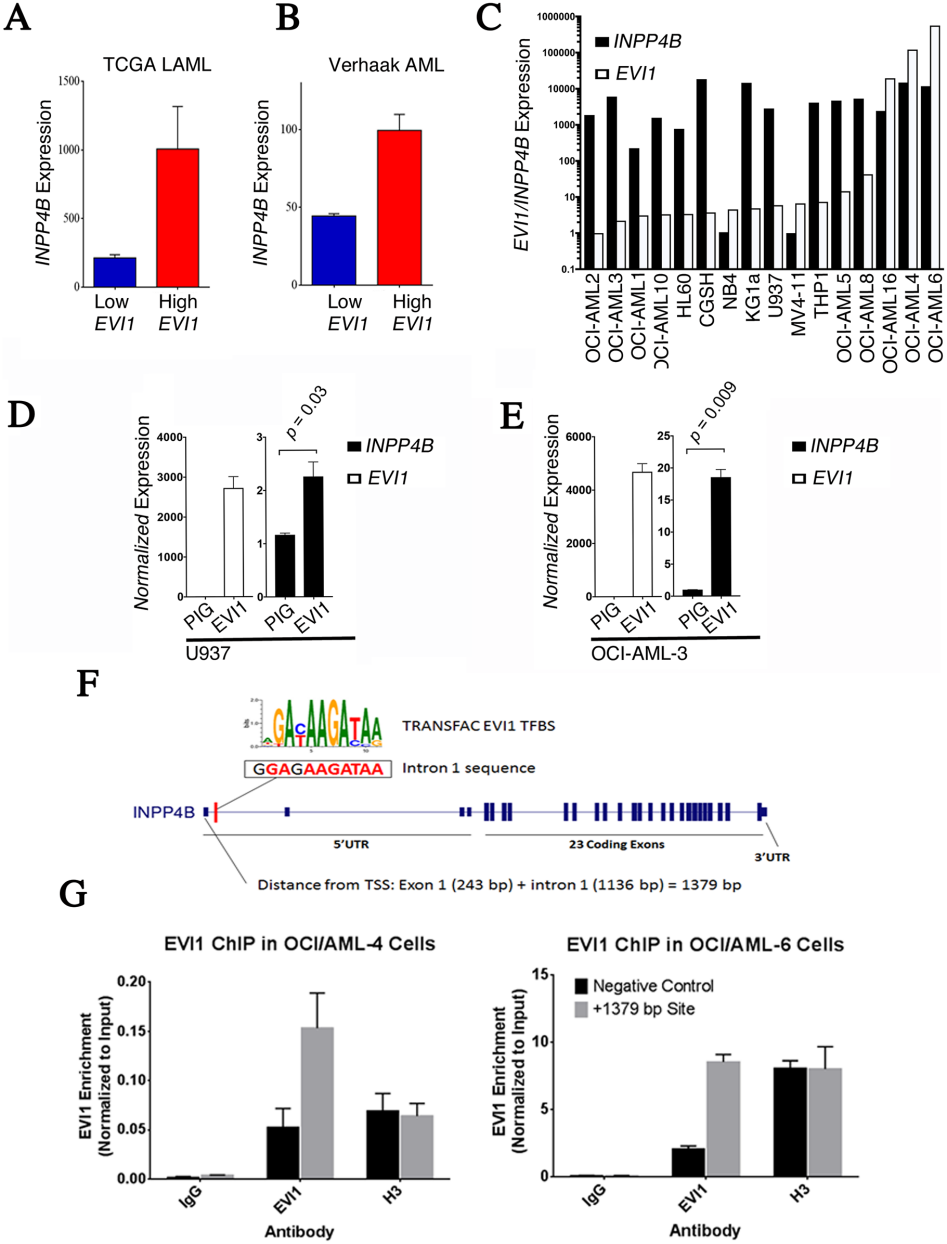
EVI1 regulates *INPP4B* expression in AML. (A) *INPP4B* expression in *EVI1*^high^ compared to *EVI1*^low^ AML patients both in TCGA-LAML and (B) Verhaak datasets. Data represent mean *INPP4B* expression in *EVI1* high vs. low groups; cutoffs were determined using Fisher’s exact optimized SubID. (C) *EVI1* and *INPP4B* expression in a panel of AML cell lines. (D,E) *EVI1* and *INPP4B* transcript expression in U937 and OCI-AML-3 cells transduced with MSCV-puro-IRES-GFP (PIG) and PIG-EVI1 retroviruses. (F) Illustration of predicted EVI1 binding site 1379bp downstream of the *INPP4B* transcription start site. (G) Anti-EVI1 ChIP in *EVI1*^*hig*h^ OCI-AML -4 and OCI-AML -6 cells demonstrated enrichment at the +1379bp EVI1 binding site of the *INPP4B* promoter region.

Due to its role in leukemogenesis and strong association with *INPP4B* expression in both Verhaak and TCGA’s LAML datasets (Fig 4A and 4B), we focused our *in vitro* validation on EVI1 to test its role as a possible regulator of *INPP4B* expression. Notably, both *INPP4B* and *EVI1* were also shown to be significantly enriched in a leukemic stem signature identified by Eppert *et al* (22). We hypothesized that the EVI1 transcription factor may regulate *INPP4B* transcription in AML cell lines. To test this, we first examined the expression levels of both *EVI1* and *INPP4B* across a panel of 16 AML cell lines (Fig 4C). We did not observe any correlation between EVI1 and INPP4B expression in these cell lines, however all cell lines with high EVI1 had high INPP4B. To determine if there was a relationship between EVI1 and INPP4B in some settings we used overexpression and knock-down studies. First, we used retroviral constructs to overexpress *EVI1* in the *EVI1*^*low*^ cell lines OCI-AML-3 and U937 where we observed significant increases in *INPP4B* expression in EVI1-infectants compared to control (Fig 4D and 4E). Next, we examined direct binding of EVI1 to the *INPP4B* promoter using chromatin immunoprecipitation (ChIP) with anti-EVI1 antibodies. EVI1-ChIP in two *EVI1*^*high*^ AML cell lines, OCI-AML-4 and OCI-AML-6, demonstrated enrichment of binding at the predicted EVI1 site at +1379bp on the *INPP4B* promoter (Fig 4F and 4G). Together these results demonstrate that the EVI1 transcription factor can regulate INPP4B expression in AML cell lines, and may be one of several transcription factors to do so. These results illustrate the usefulness of SubID to identify the association of INPP4B and other transcription factors which may increase INPP4B expression in an EVI1 independent manner.

### SubID identifies prognostic significance of *INPP4B* across cancers

Despite emerging evidence demonstrating the prognostic value of *INPP4B* expression levels in some cancers, the prognostic value of *INPP4B* gene expression across many cancers remains unknown. Since gene expression-based biomarkers may offer a rapid and cost effective method for risk stratification, we assessed whether *INPP4B* gene expression is associated with patient outcome across many cancers by applying CoxPH SubID to *INPP4B* gene expression and clinical data from 25 cancer datasets from TCGA (Table 1, S1 Table). The gene expression datasets ranged in size from 119 to 1083 patients, and encompassed an assortment of different cancer types. In each cancer, SubID was used to calculate a CoxPH *P* value for every percentile cut-off point. Only cut-offs between 10% to 90% based were considered due to small subgroups sizes and statistical limitations outside of this range. As before, the minimum CoxPH *P* value yielded the maximal difference in survival statistics between the *INPP4B*^low^ and *INPP4B*^high^ group, and thus was defined as the optimal cut-off point for population dichotomization in the examined datasets. Both FDR at the optimal cut-off point (FDR_A.C._), and FDR of the 10–90% range of permutated data (FDR_10-90%_) were also calculated.

**Table 1:** Cancers with prognostically significant *INPP4B* expression status.

Cancer Type	Code	n =	Optimal_10-90%_				Median			
			%	HR	*P* Value	FDR_A.C._	HR	*P* Value	FDR	FDR_10-90%_
Kidney Renal Clear Cell Carcinoma	KIRC	531	30%	1.94	1.71E-05	1.00E-03	1.56	3.97E-03	4.00E-03	1.00E-03
Bladder Urothelial Carcinoma	BLCA	404	10%^¥^	2.27	5.62E-05	1.00E-03	1.00	9.78E-01	9.78E-01	1.00E-03
Pancreatic Adenocarcinoma	PAAD	178	40%	0.38	8.72E-05	1.00E-03	0.47	5.44E-04	4.00E-03	3.00E-03
Liver Hepatocellular Carcinoma	LIHC	370	18%	2.13	4.44E-04	2.00E-03	1.73	2.29E-03	3.00E-03	1.10E-02
Head and Neck Squamous Cell Carcinoma	HNSC	518	86%	0.60	5.22E-03	6.00E-03	0.81	1.29E-01	1.28E-01	6.70E-02
Lung Adenocarcinoma	LUAD	507	75%	0.65	7.96E-03	1.20E-02	0.90	4.91E-01	4.86E-01	8.80E-02
Skin Cutaneous Melanoma	SKCM	455	69%*	0.69	8.56E-03	9.00E-03	0.86	2.54E-01	2.39E-01	1.14E-01
Stomach Adenocarcinoma	STAD	411	21%	0.58	1.44E-02	1.10E-02	0.92	6.02E-01	6.07E-01	1.51E-01
Lung Squamous Cell Carcinoma	LUSC	494	70%*	0.70	1.54E-02	1.80E-02	0.75	3.44E-02	3.30E-02	1.95E-01
Acute Myeloid Leukemia	LAML	161	76%	0.60	2.24E-02	1.90E-02	0.83	3.59E-01	3.71E-01	2.38E-01
Kidney Renal Papillary Cell Carcinoma	KIRP	289	60%	0.52	3.44E-02	3.30E-02	0.63	1.33E-01	1.42E-01	3.03E-01
Cervical Squamous Cell Carcinoma and Endocervical Adenocarcinoma	CESC	304	48%*	0.61	4.76E-02	5.10E-02	0.69	1.31E-01	7.60E-02	3.76E-01
Brain Lower Grade Glioma	LGG	511	61%*	1.46	4.82E-02	3.80E-02	1.08	6.81E-01	6.73E-01	3.47E-01

* Optimal cutoff outside of 10–90% range, secondary optimal cutoff used.

^¥^ Small (n<50) subgroup. Significant *P* and FDR values shaded in gray

Pan-cancer analysis with CoxPH SubID revealed that *INPP4B* expression status was significantly associated with patient survival in 13 cancers (*P*<0.05, FDR_A.C_<0.05) (Table 1). Low levels of *INPP4B* expression (*INPP4B*^low^) in 8/13 cancers were associated with favourable prognosis cancers, whereas 5/13 cancers were associated with poor prognosis. The impact of *INPP4B* expression has been previously reported in four of these cancers: bladder cancer (23), lung cancer (24), melanoma (12,20), and AML [17,18].

**Fig 5:**
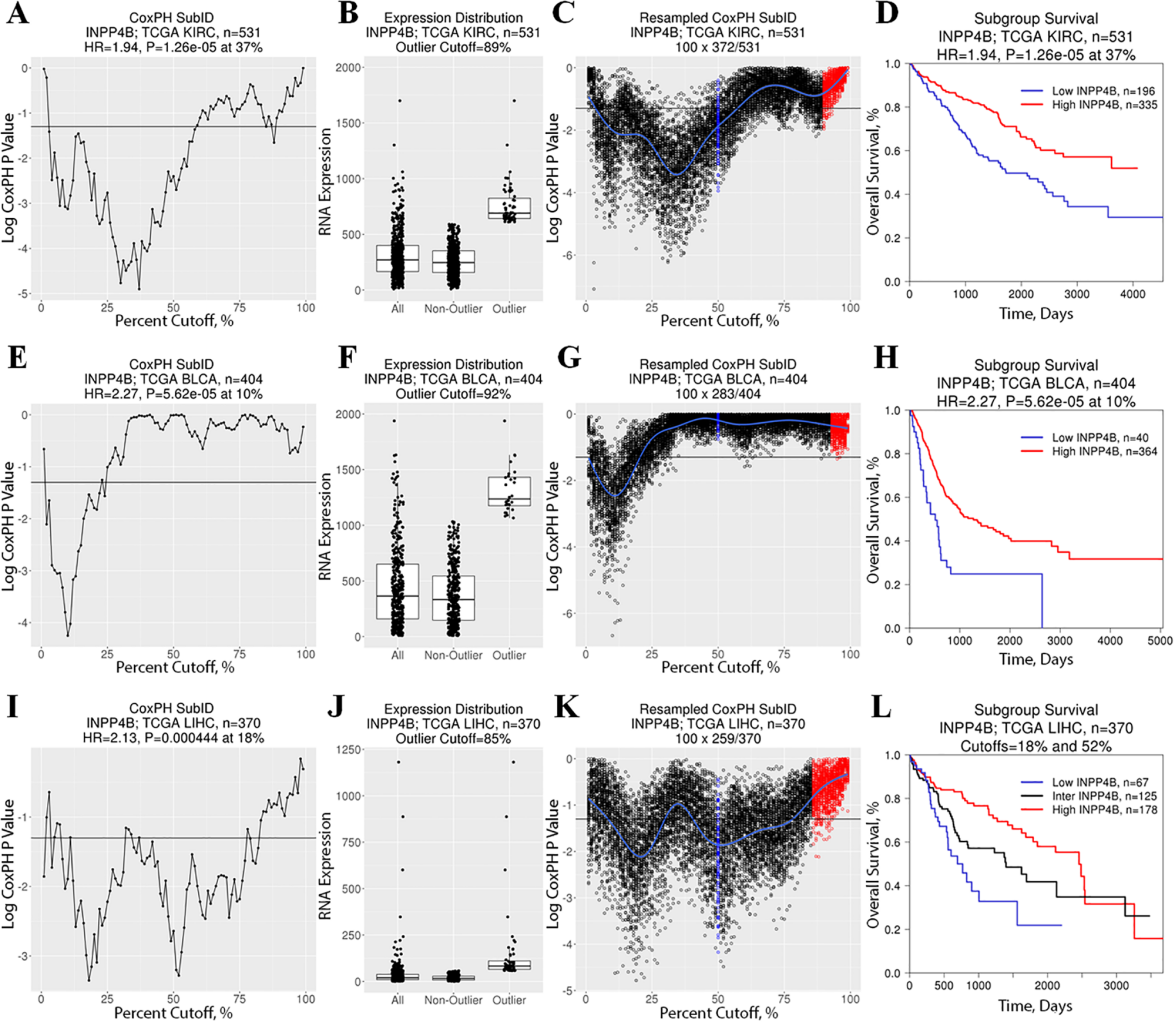
Pan-cancer *INPP4B* prognostic significance. Standard CoxPH SubID output consisting of (A,E,I) SubID plot, (B,F,J) *INPP4B* expression boxplot with expression distribution outlier status, and (C,G,K) resampled SubID plot with median (blue) and outlier cut-off values (based on expression distribution; red) for (A-D) kidney clear cell carcinoma, (E-H) bladder urothelial carcinoma, and (I-L) liver hepatocellular carcinoma reveals prognostically significant association between *INPP4B* expression status and patient survival. Specifically, *INPP4B*^low^ is associated with shorter overall survival in (D) kidney, (H) bladder and (L) liver cancers. Furthermore, *INPP4B* expression status defines three prognostically distinct subgroups within liver cancer.

Using our very stringent parameters to eliminate any false positives, only four datasets had a significant (<0.05) FDR_10-90%_. Of these, three were also significant at median cut-off. Notably in AML, a context where *INPP4B* has been previously shown to be prognostically significant, did not pass the FDR_10-90%_ significance threshold. Specifically, *INPP4B*^low^ status was shown to be associated with shorter survival (consistent with tumor suppressive function) in TCGA kidney clear cell (Fig 5A–5D; S4A, S4B and S5A Figs), bladder urothelial (Fig 5E–5H; S4C, S4D and S5B Figs), and liver hepatocellular (Fig 5I–5L; S4E, S4F and S5C Figs) carcinoma datasets. In liver hepatocellular carcinoma, INPP4B expression levels defined three distinct prognostic subgroups (*INPP4B*^low^, *INPP4B*^inter^, and *INPP4B*^high^).

SubID identified that *INPP4B* expression was significantly associated with patient survival in 13 of 25 datasets (*P*<0.05, FDR_A.C._<0.05), with significant cut-offs ranging from 10% to 86%. By contrast, examination of *INPP4B* prognostic significance using a median cut-off in these datasets, revealed prognostic significance in only four datasets. In all 13 datasets, the SubID optimized cut-off was associated with a lower *P* value and greater HR than identified by the median cut-off (the directionality of HR was the same in both median and optimal cut-offs for all cancers). Thus, consistent with its intended function, these results demonstrate that SubID outperforms median dichotomization in identifying prognostically distinct subgroups and establishing a gene expression cut-off for prognostic assessment.

**Fig 6:**
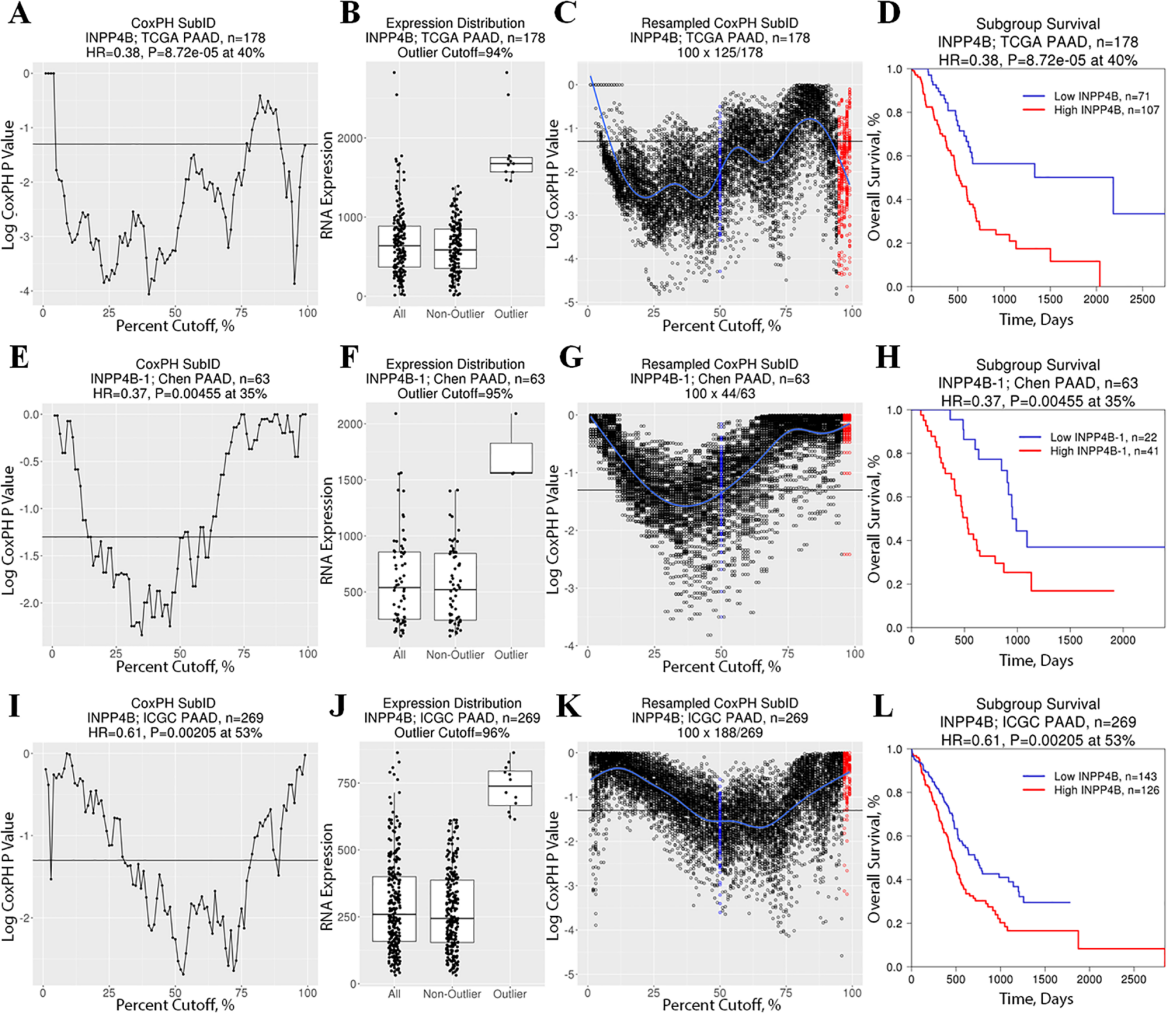
*INPP4B*^high^ is associated with poor outcome in pancreatic cancer. Standard CoxPH SubID output consisting of (A,E,I) SubID plot, (B,F,J) *INPP4B* expression boxplot with expression distribution outlier status, and (C,G,K) resampled SubID plot with median (blue) and outlier cut-off values (based on expression distribution; red) for (A-D) TCGA (E-H) Chen, and (I-L) ICGC pancreatic cancer datasets reveals prognostically significant association between *INPP4B* expression status and patient survival. Specifically, *INPP4B*^high^ is associated with shorter overall survival across all three datasets with a reoccurring optimal cut-off at 35–53% range.

Conversely, *INPP4B*^low^ status was associated with longer patient survival in pancreatic adenocarcinoma suggesting an oncogene-like, rather than a tumor suppressor-like, clinical association (Fig 6A–6D; S6A, S6B and S7A Figs). Taking advantage of independent pancreatic cancer gene expression datasets with survival data, we performed cross-validation to validate the findings observed in TCGA. Similar to TCGA, *INPP4B*^high^ was shown to be associated with shorter overall survival both in the Chen (Fig 6E–6H; S6C, S6D and S7B Figs) and ICGC (Fig 6I–6L; S6E, S6F and S7C Figs) datasets. Furthermore, the SubID-identified optimal cut-off is applicable to all three datasets with optimal cut-offs ranging from 35% to 53%.

Together, CoxPH SubID analysis identified three previously unreported contexts where *INPP4B* expression status was shown to be prognostically important: kidney clear cell, liver hepatocellular, and bladder urothelial carcinomas. However, while *INPP4B*^low^ was found to be associated with shorter patient survival in these three contexts, *INPP4B*^low^ was associated with longer survival in pancreatic adenocarcinoma. Furthermore, the prognostic significance of *INPP4B* in pancreatic adenocarcinoma was validated in two additional independent pancreatic adenocarcinoma datasets. Survival analysis in all these six datasets once again demonstrated SubID’s superior ability to identify and isolate prognostically distinct patients based on gene expression of a prognostically informative gene in comparison to median dichotomization (S5 and S7 Figs).

## Discussion

In this study we introduce and describe the development and application of SubID, a non-median dichotomization tool developed to study heterogeneous populations. As part of its optimal cut-off mapping pipeline, SubID provides the user with information that can identify the presence of distinct subgroups based on a dichotomization variable (e.g. gene expression) and user-specified optimization parameter (e.g. survival metric or co-expression). In prognostic studies, CoxPH SubID can significantly improve patient risk stratification over median dichotomization for improved prediction of survival, which may in turn guide selection of best treatment course for patients. In co-expression studies, Fisher’s exact SubID also provides an improved assessment of co-expression with a user-specified optimal association point. Practically, SubID allows for input of any continuous variable and user specified optimization parameter. Thus, while this study describes the use of gene expression as the continuous variable, and Fisher’s exact or CoxPH *P* values as the optimization parameters, other input data can be accommodated as well. SubID was developed in recognition that populations are not homogenous, and that the relationship between a continuous variable and output parameter may be complex, rather than direct. In other words, SubID can identify small yet significant subgroups with the population, as well as it can identify the presence of multiple distinct yet significant subgroups. Furthermore, SubID improves upon other non-median dichotomization strategies with is its assessment of FDRs using data permutations and resampling steps. Specifically, SubID relies on two separate FDR analyses (FDR_A.C._ and FDR_range_) increase the confidence of the identified association with clinical outcome.

Following development and validation of previous investigations in AML [17], Fisher’s exact SubID was used to identify the top six DNA-binding transcription factors co-expressed with *INPP4B* in AML. Though all six top transcription factors (*TCF4*, *NFATC2*, *KLF12*, *GATA3*, *STAT4*, and *EVI1*) were highly associated with *INPP4B* expression, particular interest was given to *EVI1* due to its known role in leukemogenesis [22]. *In vitro* examination demonstrated that overexpression of EVI1 leads to elevated INPP4B, thus providing a rational to investigate a direct relationship between EVI1 and INPP4B. Chromatin immunoprecipitation demonstrated direct physical binding of EVI to the *INPP4B* transcription start site region. Together, these experimental results support a role for EVI1 in regulating *INPP4B* expression in AML and consequently providing validation of SubID’s ability to identify co-expressed genes.

In addition to uncovering EVI1 as transcription factor regulating *INPP4B* expression, SubID was also used to examine the prognostic significance of *INPP4B* expression status across cancers. Previous studies demonstrated the prognostic significance of INPP4B protein loss or overexpression across some different cancer types [25]. Despite its function being poorly understood, emerging data suggested that INPP4B may have a context dependent role in cancer [25]. Mechanistically, the “canonical” tumour suppressive mechanism of INPP4B functions through its regulation of Akt-activity [9]. On the other hand, a description of an oncogene-like role for INPP4B can be explained through its ability to activate SGK-3, a PI(3)P regulated kinase [23]. Interestingly, Akt and SGK-3 kinases share many substrates which suggests that whether INPP4B is high or low, the same downstream pathways may be activated including GSK3*β*, PRAS40, TSC1/2, FOXO3A, BAD [26]. Nevertheless, more mechanistic studies will solidify our understanding of this contextual role for INPP4B in cancer (S8 Fig).

A more comprehensive characterization of *INPP4B*’s prognostic value across cancers could provide a starting point for studies examining a mechanism responsible for INPP4B’s observed context-dependent nature. Thus, in this study, we systematically examined the prognostic significance of *INPP4B* expression status across all TCGA datasets from 25 different cancer types. CoxPH SubID application to our pan-cancer study identified 13 cancers where *INPP4B* expression status was prognostically significant. The association of *INPP4B* with survival was in-line with previously reported tumor suppressive function in bladder cancer, and putative tumor-promoting role in melanoma and AML [12,17,18,23]. However, our findings in lung cancer contradict those described in the literature. Specifically, while Zhang *et al*. demonstrated that INPP4B suppresses proliferation, colony formation potential and anchorage-independent growth in lung cancer [24], our clinical findings indicate high levels of INPP4B expression are associated with poor patient outcome. It is important to note however, that only bladder cancer is part of the four cancers which met subsequent FDR_10-90%_<0.05 cut-off, and are hence high confidence hits. Thus, our findings confirm the known tumor-suppressive role of *INPP4B* in bladder cancer, and uncover three new contexts where *INPP4B* expression status may be prognostically significant. Notably, our findings are the first to provide evidence demonstrating an association between *INPP4B*^high^ and shorter overall survival in pancreatic adenocarcinoma. This oncogene-like association with patient outcome was observed in three independent datasets, and is reminiscent of our observations in AML. Thus, the pan-cancer analysis once again reveals the opposing relationship between *INPP4B* expression and patient outcome. Consistent with literature, this systematic analysis reveals that the relationship between *INPP4B* and cancer survival is context dependent.

To examine the relationship between *INPP4B* expression status and clinical outcome, both optimal and median cut-offs were examined. While the median cut-off generally has superior power—by maximizing the size of the compared subgroups—it does so at the expense of specificity. Some patient subgroups may account for only a small proportion of the population, thus to potentially identify those subgroups, the population must be dichotomized to separate the subgroup from the rest of the population if possible. The underlying basis of SubID relies on the notion that specific genes can be tied to a specific subgroup of patients. Thus, by identifying these genes and identifying the expression level which characterizes the subgroup of interest, we can achieve patient subgrouping which is superior to the more commonly used median subgrouping. Due to our non-median dichotomization approach, we were able to identify clinically distinct subgroups accounting for <50% of the patients based on their *INPP4B* expression status. This approach has also allowed us to identify additional datasets where *INPP4B* expression status is clinically significant, which were not picked up using median dichotomization.

Finally, application of the SubID subgroup identification pipeline to study the role of *INPP4B* across cancers revealed the context dependent nature of *INPP4B*. Depending on the context, *INPP4B*^low^ can be associated with either shorter or longer patient survival. Additionally, *INPP4B* expression can be used to stratify patients into two or three distinct subgroups as was observed in liver hepatocellular carcinoma with *INPP4B*^low^, *INPP4B*^inter^, and *INPP4B*^high^ subgroups. Furthermore, the analysis pipeline described here provides a valuable strategy for studying the relationship between gene expression and clinical outcome. Such improved stratification can be used to better predict patient outcome, and thus adjust the therapeutic regimen to improve patient survival. It is important to note however that though the goal of FDR correction is to minimize the likelihood of false positives, the ultimate “FDR” assessment remains cross-validation in multiple independent datasets backup up with experimental results. Thus, a non-significant FDR_range_ does not mean that *INPP4B* expression status is not significant in that dataset. A specific example is the AML dataset, which while it did not achieve FDR_10-90%_ significance, has been validated in multiple datasets in previous studies [17,18]. However, our development of SubID provides a major step away from the use of median dichotomization for patient stratification, a detriment which ignores patient heterogeneity and the existence of multiple and/or small distinct subgroups with a population.

## Supporting information

S1 Fig *INPP4B* expression distribution in AML. *INPP4B* expression across patients of the (A,B) Verhaak, (C,D) TCGA, and (E,F) OCI/PM AML datasets as visualized by a (A,C,E) bar plot and a (B,D,F) density plot.
(TIF)

S2 Fig *INPP4B* prognostic significance in AML based on median dichotomization. (A) Verhaak, (B) TCGA, and (C) OCI/PM AML dataset patient survival based on median dichotomization of *INPP4B* expression.
(TIF)

S3 Fig Potential transcriptional regulators of *INPP4B* (TCGA LAML dataset). Color and status (based on Verhaak dataset-derived maximal co-expression analysis) heatmaps of the top potential transcriptional regulators of INPP4B expression in AML (TCGA AML dataset).
(TIF)

S4 Fig Pan-cancer *INPP4B* expression distribution. *INPP4B* expression across patients of the TCGA (A,B) kidney clear cell carcinoma, (C,D) bladder urothelial carcinoma, and (E,F) liver hepatocellular carcinoma datasets visualized by a (A,C,E) bar plot and a (B,D,F) density plot.
(TIF)

S5 Fig Pan-cancer *INPP4B* prognostic significance based on median dichotomization. TCGA (A) kidney clear cell carcinoma, (B) bladder urothelial carcinoma, and (C) liver hepatocellular carcinoma dataset patient survival based on median dichotomization of *INPP4B* expression.
(TIF)

S6 Fig *INPP4B* expression distribution in pancreatic adenocarcinoma. *INPP4B* expression across pancreatic adenocarcinoma patients of the (A,B) TCGA, (C,D) Chen, and (E,F) ICGC datasets as visualized by a (A,C,E) bar plot and a (B,D,F) density plot.
(TIF)

S7 Fig *INPP4B* prognostic significance in pancreatic adenocarcinoma based on median dichotomization. Pancreatic adenocarcinoma (A) TCGA, (B) Chen, and (C) ICGC dataset patient survival based on median dichotomization of *INPP4B* expression.
(TIF)

S8 Fig INPP4B overexpression signals through SGK3 which phosphorylates many of the same targets as AKT. SGK3 is recruited to the cell membrane by PI(3)P and activated by PDK1 phosphorylation. The sequence similarity allows both AKT and SGK3 to phosphorylate the same RXRXXS/T motif, thus allowing them to target many of the same downstream substrates.
(TIF)

S1 Table Datasets with no significant cut-off within the 10 to 90% range. (PDF)
