# Correction: SubID, a non-median dichotomization tool for heterogeneous populations, reveals the pan-cancer significance of *INPP4B* and its regulation by EVI1 in AML

**DOI:** 10.1371/journal.pone.0195417

**Published:** 2018-03-29

**Authors:** The PLOS ONE Staff

There is information missing from the Funding section. The correct funding information is as follows: This work was funded by Canada Research Chair funds to LS and Leukemia and Lymphoma Society of Canada Grant (#317359) to LS, the Orsino Chair in Leukemia Research, Marvin and Linda Barnett leukemia Research Fund, Brian Steck Leukemia Research Fund to MDM and an Innovation Grant of the Canadian Cancer Society, and Brain Canada with the financial support of Health Canada (# 705166) to JR. The funders had no role in study design, data collection and analysis, decision to publish, or preparation of the manuscript.

The publisher apologizes for the error.
