# Supplementary figures and images for: SubID, a non-median dichotomization tool for heterogeneous populations, reveals the pan-cancer significance of INPP4B and its regulation by EVI1 in AML
Source: PLoS One. 2018 Feb 7;13(2):e0191510. doi: 10.1371/journal.pone.0191510 (PMC5802890; doi:10.1371/journal.pone.0191510)

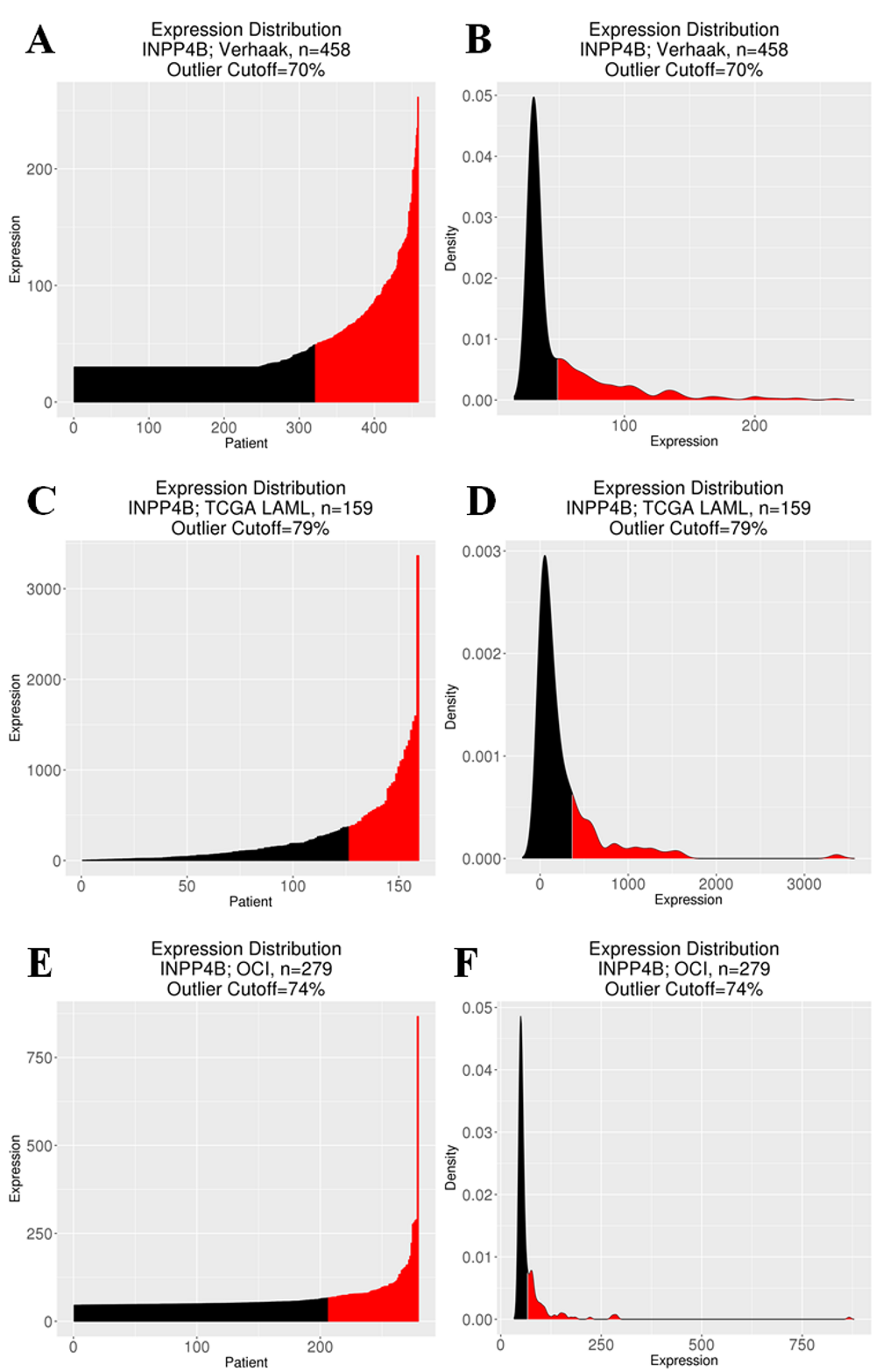

Supplement: S1 Fig — INPP4B expression across patients of the (A,B) Verhaak, (C,D) TCGA, and (E,F) OCI/PM AML datasets as visualized by a (A,C,E) bar plot and a (B,D,F) density plot. (TIF) [file pone.0191510.s001.tif]

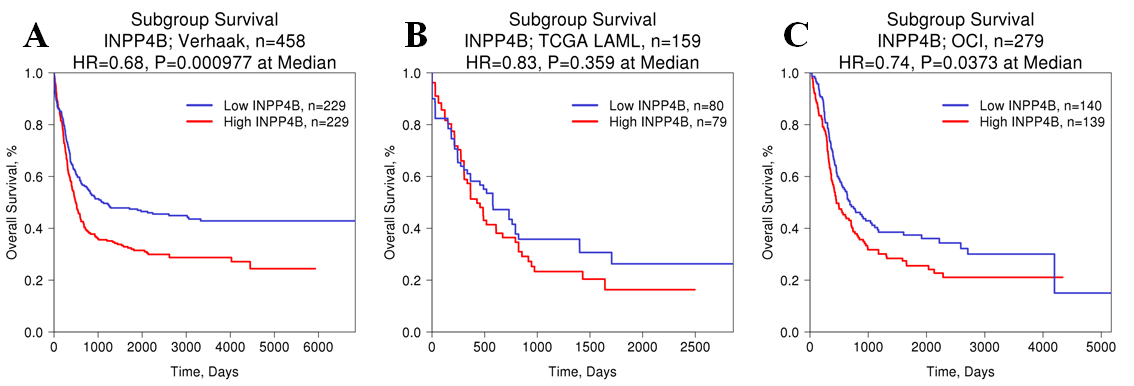

Supplement: S2 Fig — (A) Verhaak, (B) TCGA, and (C) OCI/PM AML dataset patient survival based on median dichotomization of INPP4B expression. (TIF) [file pone.0191510.s002.tif]

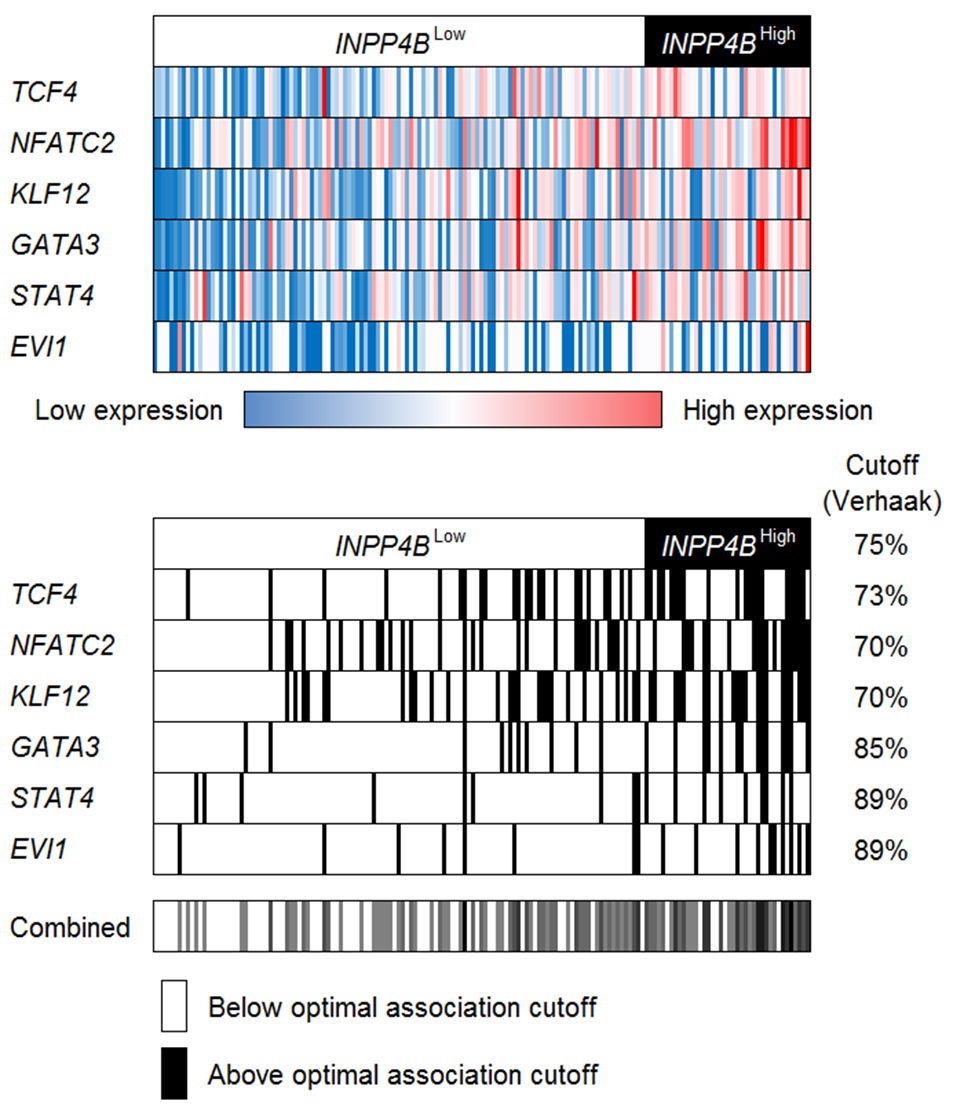

Supplement: S3 Fig — Color and status (based on Verhaak dataset-derived maximal co-expression analysis) heatmaps of the top potential transcriptional regulators of INPP4B expression in AML (TCGA AML dataset). (TIF) [file pone.0191510.s003.tif]

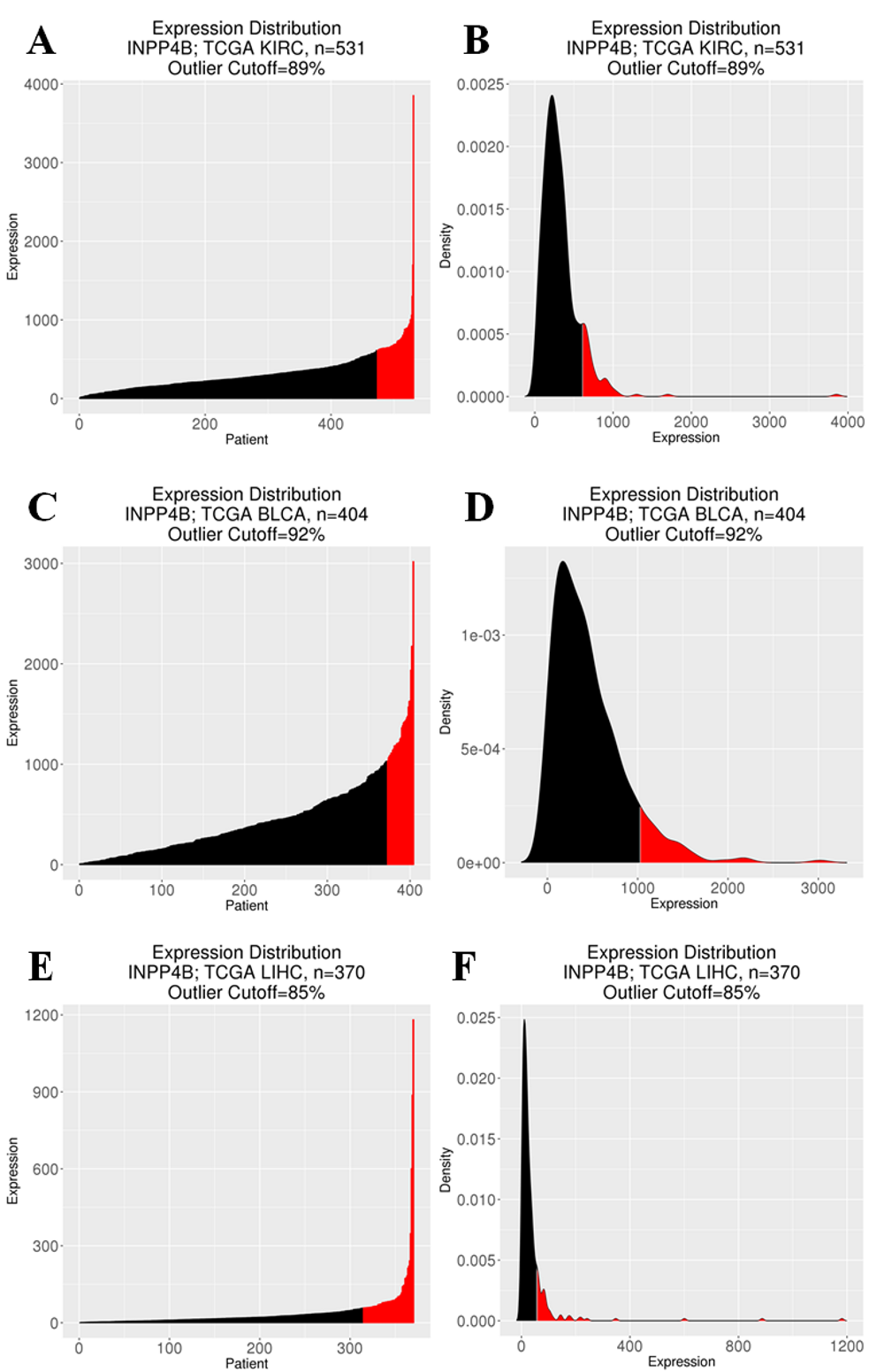

Supplement: S4 Fig — INPP4B expression across patients of the TCGA (A,B) kidney clear cell carcinoma, (C,D) bladder urothelial carcinoma, and (E,F) liver hepatocellular carcinoma datasets visualized by a (A,C,E) bar plot and a (B,D,F) density plot. (TIF) [file pone.0191510.s004.tif]

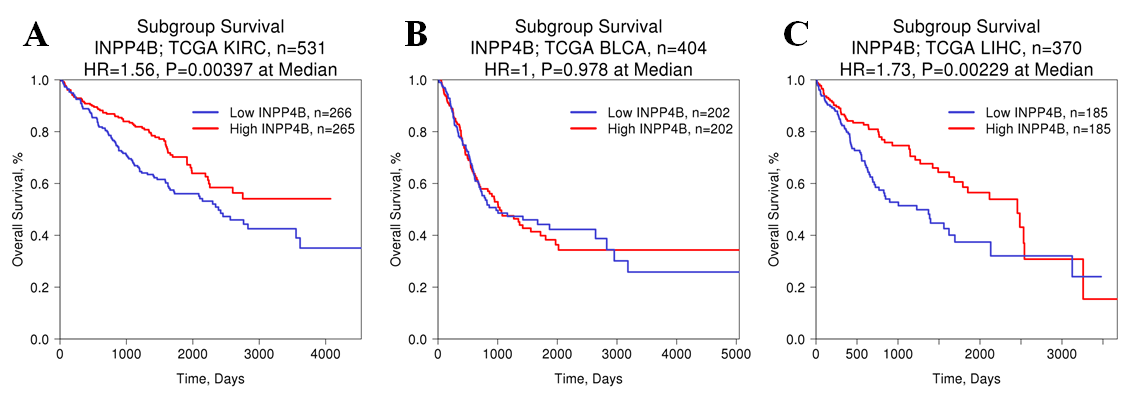

Supplement: S5 Fig — TCGA (A) kidney clear cell carcinoma, (B) bladder urothelial carcinoma, and (C) liver hepatocellular carcinoma dataset patient survival based on median dichotomization of INPP4B expression. (TIF) [file pone.0191510.s005.tif]

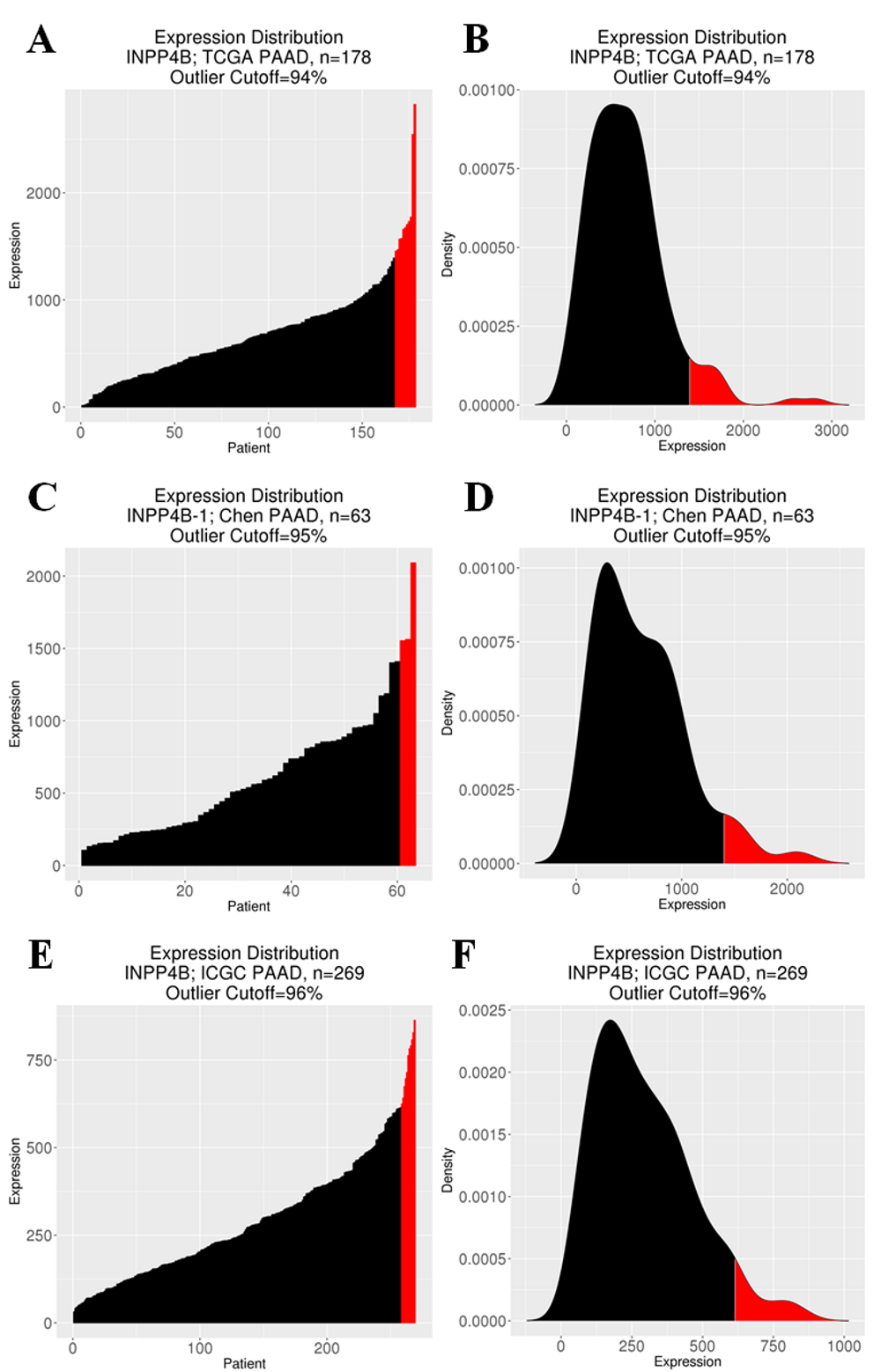

Supplement: S6 Fig — INPP4B expression across pancreatic adenocarcinoma patients of the (A,B) TCGA, (C,D) Chen, and (E,F) ICGC datasets as visualized by a (A,C,E) bar plot and a (B,D,F) density plot. (TIF) [file pone.0191510.s006.tif]

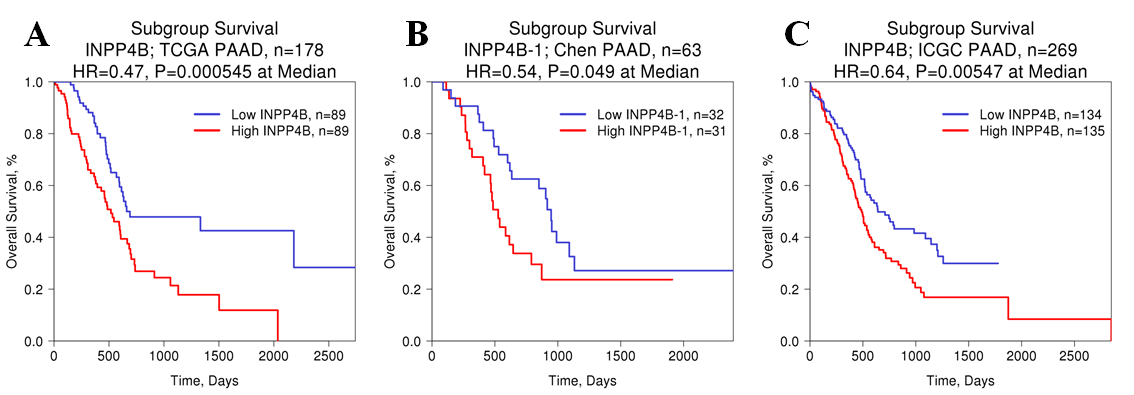

Supplement: S7 Fig — Pancreatic adenocarcinoma (A) TCGA, (B) Chen, and (C) ICGC dataset patient survival based on median dichotomization of INPP4B expression. (TIF) [file pone.0191510.s007.tif]

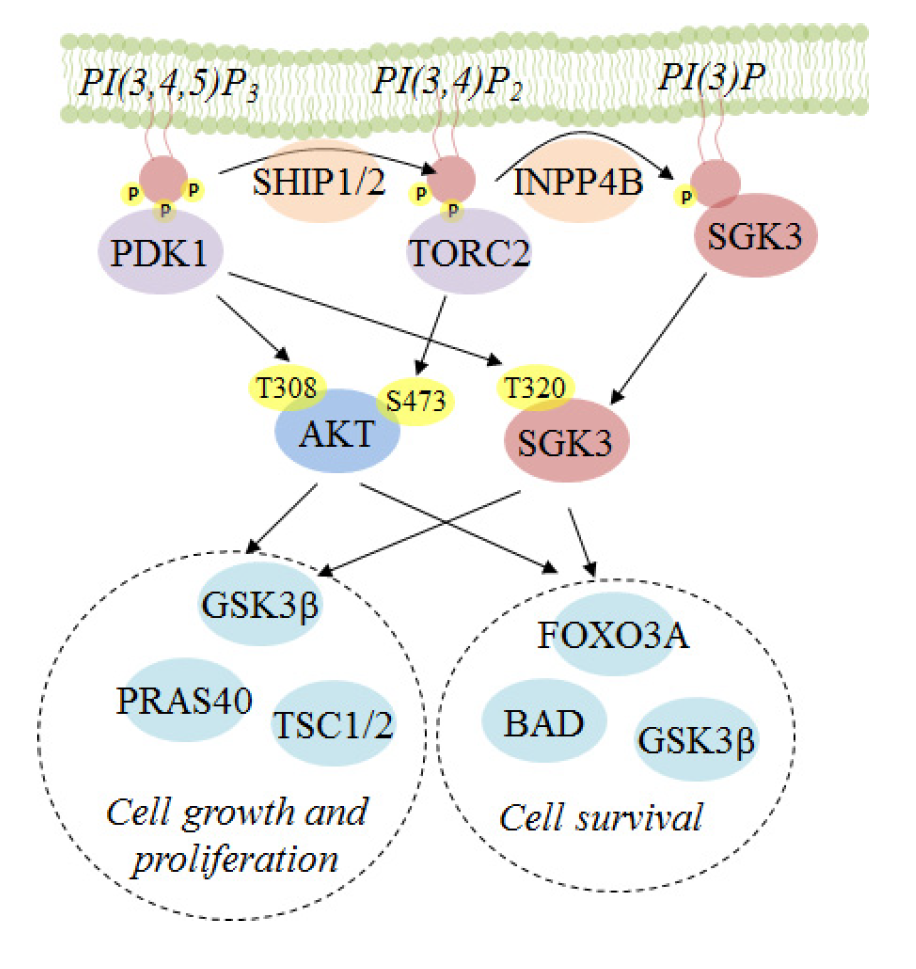

Supplement: S8 Fig — SGK3 is recruited to the cell membrane by PI(3)P and activated by PDK1 phosphorylation. The sequence similarity allows both AKT and SGK3 to phosphorylate the same RXRXXS/T motif, thus allowing them to target many of the same downstream substrates. (TIF) [file pone.0191510.s008.tif]
