# Supplementary material for: SubID, a non-median dichotomization tool for heterogeneous populations, reveals the pan-cancer significance of INPP4B and its regulation by EVI1 in AML
Source: PLoS One. 2018 Feb 7;13(2):e0191510. doi: 10.1371/journal.pone.0191510 (PMC5802890; doi:10.1371/journal.pone.0191510)
Supplement: S1 Table — (PDF) [file pone.0191510.s009.pdf]

Table S1. Datasets with no significant cut-off within the 10 to 90% range

Cancer Type	Code	n=	Optimal$_{10\text{-}90\%}$				Median		
			%	HR	*P* Value	FDR$_{A.C.}$	HR	*P* Value	FDR$_{A.C.}$
Glioblastoma Multiforme	GBM	555	66%	0.84	7.04E-02	6.60E-02	0.90	2.61E-01	2.51E-01
Breast Invasive Carcinoma	BRCA	1083	54%*	1.34	8.27E-02	9.20E-02	1.13	4.53E-01	3.07E-01
Thyroid Carcinoma	THCA	505	89%	0.38	9.22E-02	6.40E-02	0.88	7.91E-01	6.44E-01
Pheochromocytoma and Paraganglioma	PCPG	178	35%	4.31	9.48E-02	2.90E-02	1.91	4.55E-01	4.13E-01
Colon Adenocarcinoma	COAD	266	15%*¥	0.51	9.66E-02	1.00E-01	0.68	1.47E-01	1.20E-01
Esophageal Carcinoma	ESCA	184	22%	1.52	1.25E-01	1.42E-01	1.04	8.52E-01	8.68E-01
Uterine Corpus Endometrial Carcinoma	UCEC	174	12%*¥	2.07	1.39E-01	9.70E-02	0.68	2.94E-01	2.76E-01
Ovarian Serous Cystadenocarcinoma	OV	502	30%	0.84	1.64E-01	1.57E-01	1.01	9.07E-01	8.97E-01
Sarcoma	SARC	259	72%	0.74	1.74E-01	1.61-01	0.98	9.32E-01	9.36E-01
Testicular Germ Cell Tumors	TGCT	134	61%*	0.20	1.90E-01	8.50E-02	0.51	5.85E-01	2.30E-01
Thymoma	THYM	119	35%	0.27	2.28E-01	1.97E-01	0.98	9.76E-01	9.48E-01
Prostate Adenocarcinoma	PRAD	497	20%*	2.80	7.39E-01	6.60E-02	1.25	7.39E-01	1.78E-01

* Optimal cutoff outside of 10-90% range, secondary optimal cutoff used. ¥ Small (n<50) subgroup. Significant *P* and FDR values shaded in gray
